# An interactive database for the investigation of high-density peptide microarray guided interaction patterns and antivenom cross-reactivity

**DOI:** 10.1371/journal.pntd.0008366

**Published:** 2020-06-24

**Authors:** Kamille E. Krause, Timothy P. Jenkins, Carina Skaarup, Mikael Engmark, Nicholas R. Casewell, Stuart Ainsworth, Bruno Lomonte, Julián Fernández, José M. Gutiérrez, Ole Lund, Andreas H. Laustsen

**Affiliations:** 1 Department of Bio and Health Informatics, Technical University of Denmark, Kongens Lyngby, Denmark; 2 Department of Biotechnology and Biomedicine, Technical University of Denmark, Kongens Lyngby, Denmark; 3 Centre for Snakebite Research & Interventions, Liverpool School of Tropical Medicine, Pembroke Place, Liverpool, United Kingdom; 4 Instituto Clodomiro Picado, Facultad de Microbiología, Universidad de Costa Rica, San José, Costa Rica; Faculty of Science, Ain Shams University (ASU), EGYPT

## Abstract

Snakebite envenoming is a major neglected tropical disease that affects millions of people every year. The only effective treatment against snakebite envenoming consists of unspecified cocktails of polyclonal antibodies purified from the plasma of immunized production animals. Currently, little data exists on the molecular interactions between venom-toxin epitopes and antivenom-antibody paratopes. To address this issue, high-density peptide microarray (hdpm) technology has recently been adapted to the field of toxinology. However, analysis of such valuable datasets requires expert understanding and, thus, complicates its broad application within the field. In the present study, we developed a user-friendly, and high-throughput web application named “Snake Toxin and Antivenom Binding Profiles” (STAB Profiles), to allow straight-forward analysis of hdpm datasets. To test our tool and evaluate its performance with a large dataset, we conducted hdpm assays using all African snake toxin protein sequences available in the UniProt database at the time of study design, together with eight commercial antivenoms in clinical use in Africa, thus representing the largest venom-antivenom dataset to date. Furthermore, we introduced a novel method for evaluating raw signals from a peptide microarray experiment and a data normalization protocol enabling intra-microarray and even inter-microarray chip comparisons. Finally, these data, alongside all the data from previous similar studies by Engmark et al., were preprocessed according to our newly developed protocol and made publicly available for download through the STAB Profiles web application (http://tropicalpharmacology.com/tools/stab-profiles/). With these data and our tool, we were able to gain key insights into toxin-antivenom interactions and were able to differentiate the ability of different antivenoms to interact with certain toxins of interest.

The data, as well as the web application, we present in this article should be of significant value to the venom-antivenom research community. Knowledge gained from our current and future analyses of this dataset carry the potential to guide the improvement and optimization of current antivenoms for maximum patient benefit, as well as aid the development of next-generation antivenoms.

## Author summary

Millions of people are bitten by venomous snakes each year, resulting in over 100,000 deaths. Currently, such envenomings are treated with animal derived antivenoms that contain undefined antibodies against snake venom toxins that have been raised by the production animal’s immune system. To date, our understanding of these antibody toxin interactions is sparse, but with the help of high-density peptide microarray (hdpm) technology this is starting to change. Whilst this technology is very powerful, analysis of the output data is complex and requires expert training. Therefore, in this study, we developed a user-friendly, and high-throughput web application named “Snake Toxin and Antivenom Binding Profiles” (STAB Profiles). Furthermore, we ensured our tool was functional and able to handle large amounts of data by creating an entirely novel and larger than ever hdpm dataset based on all African snake toxin proteins together with eight commercial antivenoms. With these data and our tool, we were able to further our understanding on toxin-antivenom interactions and were able to differentiate the ability of different antivenoms to interact with certain toxins of interest. Ideally, these and future insights can help guide the improvement and optimization of current antivenoms, as well as aid the informed development of next-generation antivenoms.

## Introduction

An urgent demand exists for addressing the global public health burden of snakebite envenoming, a neglected tropical disease that each year exacts a death toll of more than 100,000 and leaves many more disfigured for life [1, 2]. When administered promptly, antivenoms derived from the plasma of hyper-immunized animals are effective in neutralizing the main clinical manifestations of snakebite envenoming, particularly the systemic effects [2–4]. Despite this, antivenoms have many limitations relating to their specificity, safety and affordability, and thus there is a strong rationale to develop new snakebite therapeutics with higher efficacy and broader species coverage, as well as at a lowered cost [5, 6]. Toxicovenomics is a proteomics-based approach that can be used to analyze snake venoms to provide an overview of which toxins are medically relevant in envenomings, and this approach shows promise for selecting the most effective venom mixtures for immunization [7–9]. However, toxicovenomics needs to be combined with complementary analytical approaches, such as animal-based neutralization assays, immunochemical studies [3], and antivenomics [10]; these, together, can provide an in-depth view into the molecular reactivity and potential neutralization of these medically relevant toxins [11, 12]. Nevertheless, all of these approaches fail to provide information about the specific binding interactions between venom toxin epitopes and antivenom antibody paratopes [11]. Such molecular interaction information is key towards developing a better understanding of the nature of antivenom cross-reactivity and para-specificity and, consequently, the development of improved and broadly neutralizing antivenoms [11, 12].

To address this issue and to facilitate high-throughput assessment of molecular interactions between venom toxin epitopes and antivenom antibody paratopes, high-density peptide microarray (hdpm) technology has recently been adapted to the field of toxinology [13–15]. Hdpms have long been successfully applied to a range of fields, such as enzyme inhibition, immunoassays, affinity agents for viruses, and therapeutic peptides amongst others [16]. However, the application of this technology to venom and antivenom research is relatively new [13]. Still, its recent introduction has already enabled the simultaneous analysis of a large number of toxins and multiple antivenoms, facilitated the identification of amino acid specific interaction sites, and provided information about shared recognition sites between homologous toxins. Indeed, Engmark et al. demonstrated how toxin epitopes recognized by antivenom antibodies could be identified for toxins from multiple mamba (*Dendroaspis* genus) and cobra (*Naja* genus) species endemic to sub-Saharan Africa [13], while a subsequent study identified and characterized linear elements of epitopes from 870 pit viper venom (*Crotalinae* subfamily) protein sequences from three major snake toxin families (snake venom metalloproteases [SVMPs], phospholipases A_2_ [PLA_2_s], and snake venom serine proteases [SVSPs]) [14]. In the most recent study, Engmark et al. identified the antibody-recognized epitope in short neurotoxin 1 from black mamba (*Dendroaspis polylepis*) venom and explored the degree of cross-reactivity across 751 other toxins from the three-finger toxin (3FTx) and dendrotoxin families [15]. Yet, whilst such studies present invaluable insights into venom-antivenom interactions, they require expert knowledge in the production and interpretation of microarray datasets, which limits the wide-spread implementation of hdpm technology in the field of toxinology and, thus, impedes furthering of our understanding of venom-antivenom interactions.

Therefore, in the present study we developed a user-friendly and high-throughput web application, named “Snake Toxin and Antivenom Binding Profiles” (STAB Profiles), to facilitate analyses of hdpm datasets. For the purpose of testing our tool and evaluating its performance with a large dataset, we conducted hdpm assays using all African snake toxin protein sequences available in the UniProt database at the time of study design, together with eight commercial antivenoms in clinical use in Africa. Furthermore, we introduced a novel method for evaluating raw signals from a peptide microarray experiment and a data normalization protocol enabling intra-microarray and even inter-microarray chip comparison. Finally, these data, alongside all the data from previous similar studies by Engmark et al. [13–15], were preprocessed according to our newly developed protocol and made publicly available for download through the STAB Profiles web application (http://tropicalpharmacology.com/tools/stab-profiles/). We hope that this interactive web application and our curated datasets can help guide the development of increasingly broadly immunoreacting and broadly neutralizing antivenoms in the near future by unravelling epitope-paratope interactions and thereby help identify which epitopes are likely the targets of neutralizing antibodies. Our data does, however, only represent the first step in such identification, which should be followed up by *in vitro* and *in vivo* validation.

### Implementation

**Fig 1:**
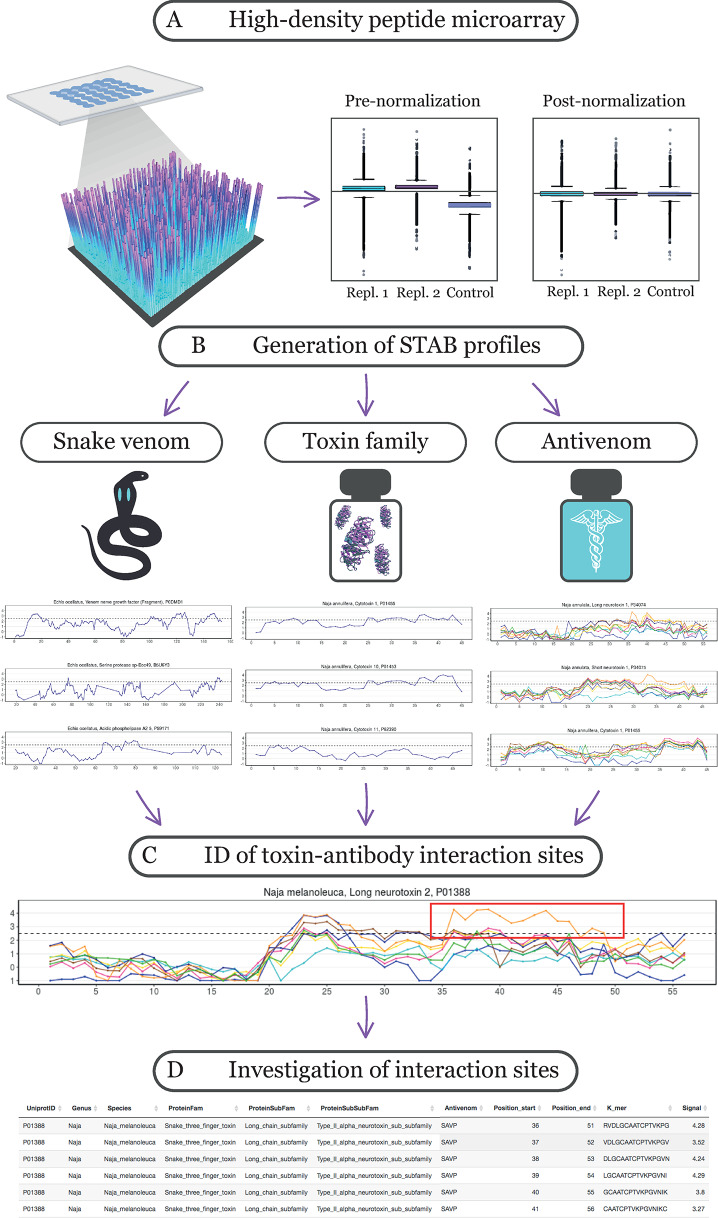
Schematic overview illustrating the creation and implementation of the Snake Toxin and Antivenom Binding Profiles (STAB Profiles) web application. (A) Initially, new high-density peptide microarray data was generated, and the signals were normalized. (B) Thereafter, we created the STAB Profiles web application and allowed for instant visualization of the data either by “snake venom”, “toxin family”, or “antivenom”. (C) This allows for the identification and selection of toxin-antibody linear interaction sites. (D) Upon selection of a region of interest the web application allows further investigation of such interaction sites and provides information about involved *k-*mers, position start/end, and signal strength amongst others.

The STAB Profiles web application has been created to provide an easy and fast visual identification and interpretation of epitope interactions between snake venom toxins and antivenom antibodies (Fig 1). It uses raw hdpm data to identify the potential presence of linear interaction sites, based on consecutive peptides producing interaction signals above a Z-score (the number of standard deviations that the signal distribution is above the noise distribution; if a Z-score is equal to 0, it is on the mean, whilst if it is equal to +1/-1, it is 1 Standard Deviation above/below the mean) of 2.5 (p < 0.05). Input data processing is further detailed in the methods section.

### Binding profile visualization–STAB Profiles

The STAB application supports the investigation of hdpm data and allows the sorting of input information by antivenom, snake genus, snake species, protein family, protein sub-family, and protein sub-subfamily. The provided antivenom selection menu is a dropdown menu, where one can select one or more antivenoms at a time and compare it/them to any snake genus present in the database. The comparisons can be further focused via the selection of specific protein sub-families detected in the specified snake genus. The resultant plots can then be sorted by snake species or protein family. Furthermore, points of interest can be selected on each plot and further investigated via the "Chosen Data Points Data Table" tab, which will provide additional information and the specific amino acid sequence represented by each point, as well as a data download function.

### Interpretation of binding profiles and epitope classification

Each binding profile plot constitutes a single UniProt amino acid sequence for a snake toxin protein or protein fragment, while every point on a graph represents the interaction signal between an antivenom antibody and a peptide. A significant pattern, indicating a potential epitope, consists of multiple consecutive peptides with a signal intensity score above the significance threshold (2.5, p < 0.05). For the evaluation of toxin-antibody interactions of multiple antivenoms, it is also of interest to investigate whether similar interaction patterns can be observed across antivenom profiles. Such consensus would further support the interpretation of putative signals as representing potentially conserved toxin-antibody interaction sites. This may possibly hold information about potential cross-reactivity of antivenom antibodies, as well as antivenom para-specificity, if combined with results from neutralization assays and antivenomics studies.

## Results

In this study, we produced the most comprehensive venom-antivenom hdpm dataset published to date and used it to test our newly developed and free to use tool, STAB Profiles. The results of this process present novel biological insights into venom-antivenom interactions, antivenom-toxin family coverage, specific linear epitope-antibody interaction sites across multiple antivenoms, and venom-antivenom interactions for medically relevant toxins.

### Description of the design of the microarray experiments

We generated hdpm data using eight polyspecific and widely distributed antivenoms against African snake species (Table 1) and a 16-mer peptide library derived from 481 toxin protein sequences (23 protein families from 40 different snake species). Following signal normalization, the STAB Profiles were generated in our web application for further analysis.

**Table 1:** Characteristics of the eight African snake antivenoms and two naïve equine serum samples used in the study.

Antivenom name	Producer	Abbreviation	Species venoms neutralized according to product insert		Active substance
			Viperidae	Elapidae	
Antivipmyn Africa	Instituto Bioclon	Bioclon: Antivipmyn	*Bitis arietans* *Bitis gabonica* *Echis leucogaster* *Echis ocellatus* *Echis pyramidum*	*Dendroaspis polylepis* *Dendroaspis viridis* *Naja haje* *Naja melanoleuca* *Naja nigricollis* *Naja pallida*	F(ab’)_2_
EchiTab-Plus-ICP	Instituto Clodomiro Picado (ICP)	ICP: EchiTab	*Bitis arietans* *Echis ocellatus*	*Naja nigricollis*	IgG
Inoserp Panafricain	Inosan Biopharma	Inosan: Inoserp	*Bitis arietans* *Bitis gabonica* *Echis leucogaster* *Echis ocellatus* *Echis pyramidum*	*Dendroaspis polylepis* *Dendroaspis jamesoni* *Naja haje* *Naja melanoleuca* *Naja nigricollis* *Naja pallida*	F(ab’)_2_
Snake Venom Antiserum (Pan Africa)	Premium Serums and Vaccines	PSV: Pan Africa	*Bitis arietans* *Bitis gabonica* *Bitis nasicornis* *Bitis rhinoceros* *Echis carinatus* *Echis leucogaster* *Echis ocellatus*	*Dendroaspis polylepis* *Dendroaspis jamesoni* *Dendroaspis angusticeps* *Dendroaspis viridis* *Naja haje* *Naja melanoleuca* *Naja nigricollis*	F(ab’)_2_
FAV-Afrique	Sanofi-Pasteur	Sanofi Pasteur: FAV	*Bitis arietans* *Bitis gabonica* *Echis leucogaster* *Echis ocellatus*	*Dendroaspis polylepis* *Dendroaspis jamesoni* *Dendroaspis viridis* *Naja haje* *Naja nigricollis*	F(ab’)_2_
SAIMR (South African Institute for Medical Research) Polyvalent Snake Antivenom	South African Vaccine Producers (SAVP)	SAVP: SAIMR	*Bitis arietans* *Bitis gabonica*	*Dendroaspis polylepis* *Dendroaspis jamesoni* *Dendroaspis angusticeps* *Hemachatus haemachatus* *Naja annulifera* *Naja melanoleuca* *Naja mossambica* *Naja nivea*	F(ab’)_2_
Snake Venom Antiserum (African)	VINS Bioproducts Limited	VINS: African	*Bitis arietans* *Bitis gabonica* *Echis leucogaster* *Echis ocellatus*	*Dendroaspis jamesoni* *Dendroaspis polylepis* *Dendroaspis viridis* *Naja haje* *Naja melanoleuca* *Naja nigricollis*	F(ab’)_2_
Snake Venom Antiserum (Central Africa)	VINS Bioproducts Limited	VINS: Central Africa	*Bitis gabonica* *Daboia russelii* *Echis carinatus*	*Dendroaspis polylepis*	F(ab’)_2_
Naïve equine serum sample 1	Instituto Clodomiro Picado (ICP)				IgG
Naïve equine serum sample 2	Instituto Clodomiro Picado (ICP)				IgG

### Snake Toxin and Antivenom Binding Profiles

#### Assessment of cross-reactivity and of *k-*mer recognition by the analyzed antivenoms

When evaluating the STAB Profiles, the first stage of analysis involved the identification of *k-*mer interaction sites (peptide sequence of the full toxin protein sequence) for antivenoms that included the venoms used in their immunization process (proxy for antivenom quality) and those that were not (e.g. potential cross-reactivity). Therefore, we expected to see higher levels of *k*-mer interaction in antivenoms that also present better *in vitro* binding and *in vivo* neutralization, whilst low signals might help identify the need for strategies towards improving the coverage of a specific antivenom.

**Fig 2:**
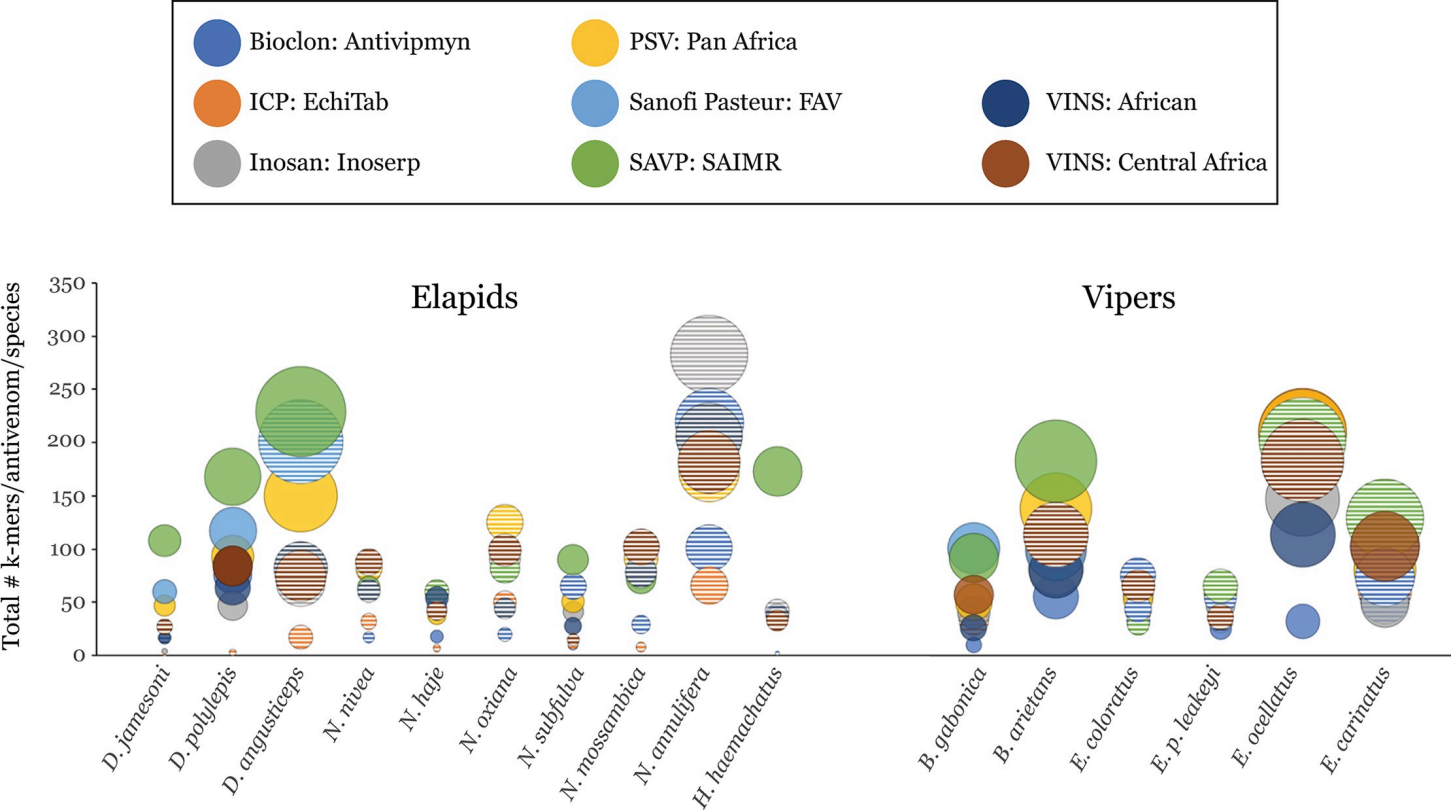
Antivenom recognition of the total number of *k-*mers above the threshold for significance for each snake species. Only venoms which have five or more toxins represented on the array are depicted. Solid circles represent venoms indicated as being effectively neutralized by an antivenom. Striped circles represent a venom which is not indicated to be neutralized by an antivenom. Bubble size is proportional to the number of toxins per species represented on the array (determined by No. of toxins × No. of *k-*mers).

We demonstrate that for most species of venomous snakes assessed, SAVP: SAIMR consistently recognized the most *k-*mers, followed by Sanofi Pasteur: FAV (Fig 1). Conversely, Bioclon: Antivipmyn contained the poorest antibody interaction across all analyzed snakes (Table 1, Fig 2). VINS: African consistently demonstrated poor binding profiles and, surprisingly, was outperformed by its regional-specific sister product, VINS: Central Africa, which demonstrated recognition of a greater number of *k-*mers in more species, despite (to the best of our knowledge) using fewer different venoms during the immunization process (Table 1, Fig 2). The regional-specific antivenom, ICP: EchiTAB, only demonstrated notable *k-*mer interaction to the vipers *Echis ocellatus* and *Bitis arietans*–two of three venoms used for immunization. Furthermore, whilst ICP: EchiTAB was superior at recognizing *E*. *ocellatus* specific *k-*mers compared to all other antivenoms, it demonstrated poor para-specific interaction to toxin *k-*mers from other *Echis* species, as well as *Bitis gabonica* (Fig 2). Conversely, SAVP: SAIMR demonstrated a substantial level of para-specific recognition towards *k-*mers derived from toxins from *E*. *ocellatus*, *E*. *carinatus*, and *E*. *p*. *leakeyi*. This was surprising, since SAVP: SAIMR was the only antivenom in this study not indicated for treating envenoming by *Echis* spp. (Fig 1C, Table 1). Furthermore, the results demonstrate that *k-*mers derived from toxins of the *Bitis* genus were recognized by all antivenoms to a lesser extent than *k-*mers from toxins from other major genera (*Dendroaspis*, *Naja*, and *Echis*) represented on the array (Fig 2). This is despite the fact that *Bitis* species toxins were represented in similar numbers as other genera (Fig 2), and that all antivenoms in this study indicate neutralization of at least one species of *Bitis* (Table 1). Notably, we also detected a large degree of para-specific elapid *k-*mer recognition by all antivenoms, which was particularly noticeable with *N*. *annulifera* and the Asian cobra *N*. *oxiana* toxin-derived *k-*mers (Fig 2). However, we also found a poor ability of all antivenoms in recognizing toxin representative *k-*mers of certain elapids, e.g. *H*. *haemachatus* (Fig 2), with the exception of SAVP: SAIMR, the only antivenom in this study indicated for treating envenoming by this species.

### Peptide interaction signal coverage of antivenoms against major families of toxin proteins

**Fig 3:**
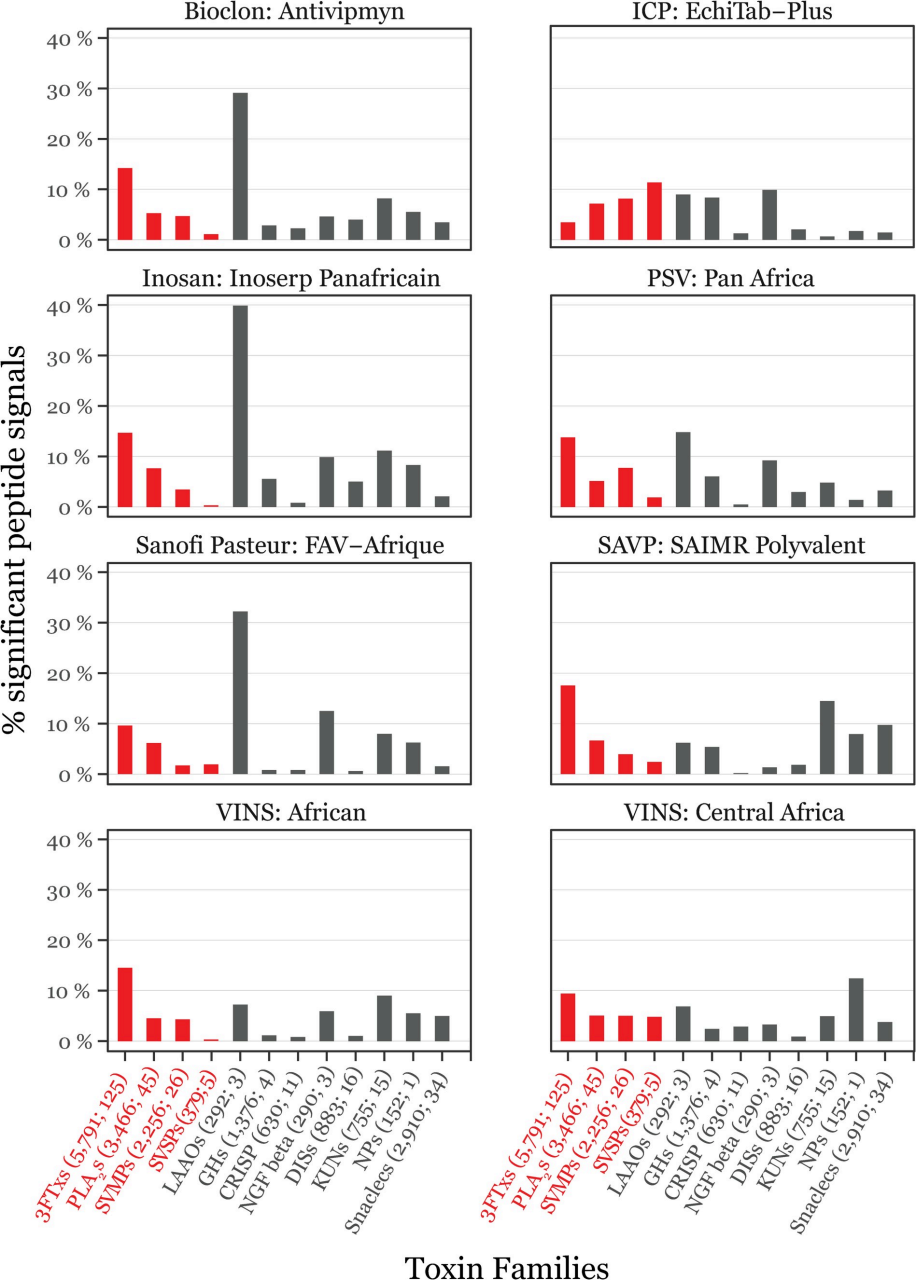
Bar graphs showing the percentages of significant 16-mer peptide binding signals per toxin family for all snakebite antivenoms tested in this study. Protein families are divided into dominant (red) and secondary (grey) toxins. The former includes three-finger toxins (3FTxs), phospholipases A_2_s (PLA_2_s), snake venom metalloproteases (SVMPs), and serine proteases (SVSPs), whereas the latter encompasses L-amino acid oxidases (LAAOs), Kunitz-type inhibitors (KUNs), cysteine-rich secretory proteins (CRISPs), glycosyl hydrolases (GHs), nerve growth factor beta (NGF beta), disintegrins (DISs), natriuretic peptides (NPs), and snake C-type lectins (Snaclecs). The numbers in brackets next to the toxin family names are the total number of 16-mer peptides used in the analysis for each protein family and the number of UniProt protein sequences the peptides per protein family were derived from.

The data enable the assessment of peptide interaction signal coverage across all eight antivenoms vs. dominant (most common and abundant across venomous snakes) and secondary (common across species, but significantly less abundant than the dominant toxin families) families of toxins [17]. Notably, out of the dominant toxin families, 3FTxs appeared to constitute the best recognized protein group (particularly cytotoxins), with peptide interaction signals for PLA_2_s and SVMPs being markedly lower (Fig 3). This is likely linked, at least in part, to the fact that 3FTxs were the most abundantly sampled group in this study. On the other hand, L-amino acid oxidases (LAAOs) and Kunitz-type inhibitors (KUNs) present the best recognized secondary toxin families, whilst very low signals were recorded for cysteine-rich secretory proteins (CRISPs) and disintegrins (DISs; Fig 3).

Out of all tested antivenoms SAVP: SAIMR demonstrated the highest peptide interaction signal coverage against 3FTxs, whilst ICP: EchiTab had the lowest (Fig 3). Conversely, ICP: EchiTab, together with Inosan: Inoserp, produced the strongest signals against PLA_2_s and, together with PSV: Pan Africa, against SVMPs (Fig 3). Meanwhile, most antivenoms produced poor interaction signals against the majority of the secondary toxin families, with only KUNs and LAAOs indicating some coverage (Fig 3).

### Binding profiles illustrate specific epitope-antibody interaction sites across multiple antivenoms

Besides generating a general understanding of the cross-reactivity potential and toxin family coverage of the tested antivenoms, this study also aimed to identify specific toxin epitopes that interact with antivenom antibodies across species and protein families. The identification of specific interaction areas of toxins could prove invaluable in the development of specific antitoxins and broadly neutralizing next-generation snakebite therapeutics.

**Fig 4:**
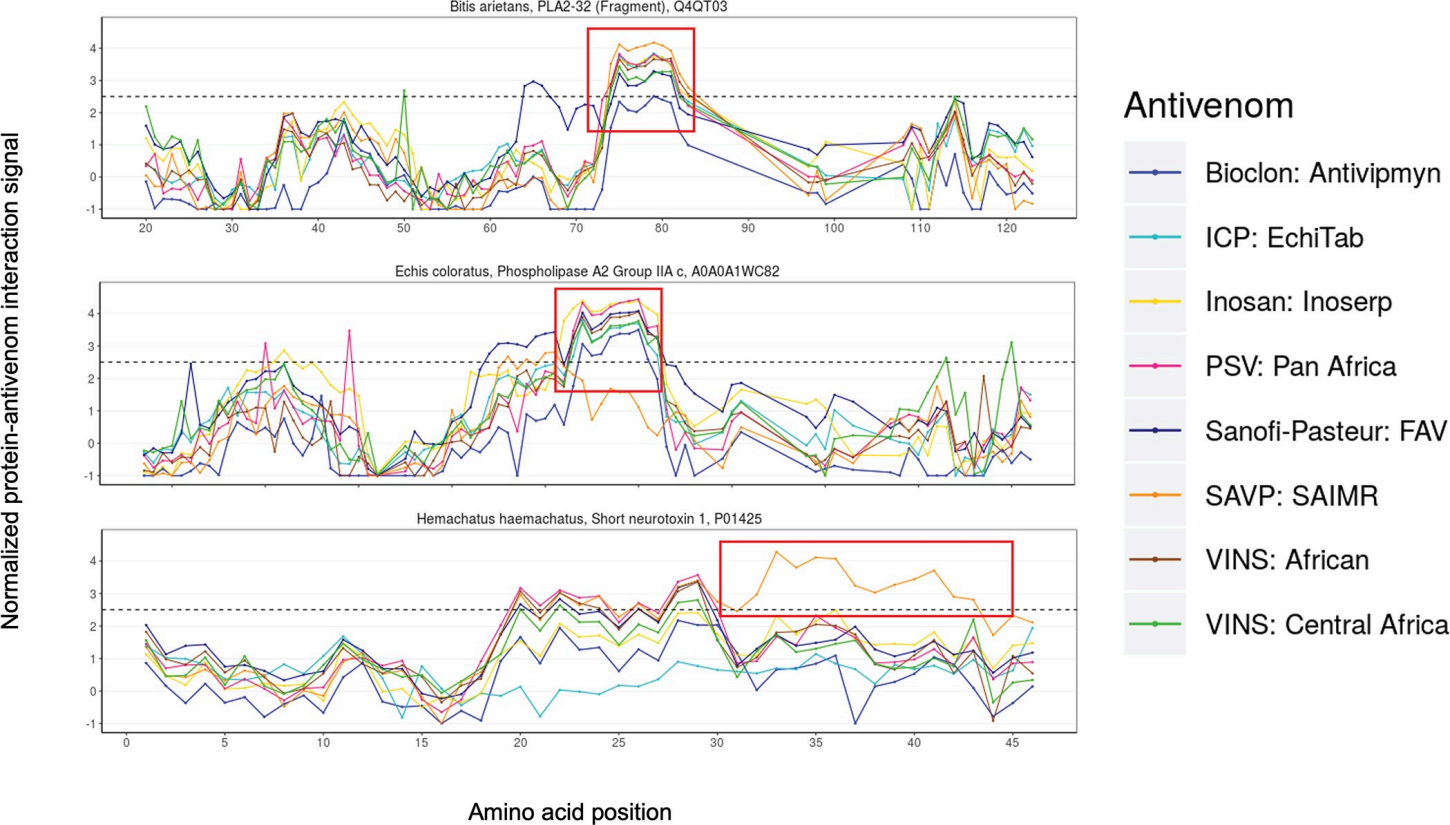
Screenshots from the STAB Profiles web application of three different types of toxin-antivenom interaction signals detected. (top) an interaction site recognized by antibodies from all antivenoms for a toxin used in the immunization mixtures (*B*. *arietans* PLA_2_ Q4QT03), (middle) an interaction site recognized by antibodies from all antivenoms for a toxin not used in the immunization mixtures (*E*. *coloratus* PLA_2_ A0A0A1WC82), and (bottom) an interaction site recognized by antibodies from only one antivenom for a toxin used in its immunization mixture *(H*. *haemachatus* short 3FTx neurotoxin P01425). Referred to sites of interaction are marked with a red square and the dotted line indicates the signal significance cutoff.

Notably, the antivenoms used in this study indicated significant and site-specific interaction signal consensus, i.e. all or the majority of antivenoms appeared to interact with the same putative epitope region of a given toxin, rather than each antivenom targeting a different binding site on the same toxin. Indeed, all tested antivenoms produced significant epitope-antibody interaction signals against a region consisting of nine subsequent *k-*mers from a PLA_2_ (UniProt ID: Q4QT03) from *Bitis arietans* (Fig 4A). An equivalent pattern of a specific epitope recognition site is also found for a PLA_2_ (UniProt ID: A0A0A1WC82) from *Echis coloratus* venom (Fig 4B). Here, all antivenoms, apart from SAVP: SAIMR, exhibited recognition of a region of 10 consecutive *k-*mers, despite none of the antivenoms using this venom as an immunogen, suggesting paraspecific epitope recognition [18]. Contrastingly, we also observed cases where antibodies from only one antivenom interacted with the target toxin. For example, a short neurotoxin (a 3FTx) from *H*. *haemachatus* venom (UniProt ID: P01425) only produced an interaction signal with SAVP: SAIMR (the only antivenom that is made with this venom as an immunogen; Fig 4C). These findings demonstrate the complexities associated with attempting to predict antibody binding interactions with often highly variable venom toxins.

### Specific epitope-antibody interaction sites across several medically relevant toxins

This study also illustrated the presence or absence of specific epitope-antibody interaction sites across a series of clinically important toxins, such as dendrotoxins, PLA_2_s, and the different 3FTx subclasses of cytotoxins, short neurotoxins, and long neurotoxins [19–21]. These data enable us to explore and predict the extent to which tested antivenoms may bind and neutralize key, medically relevant toxins.

### Dendrotoxins

**Fig 5:**
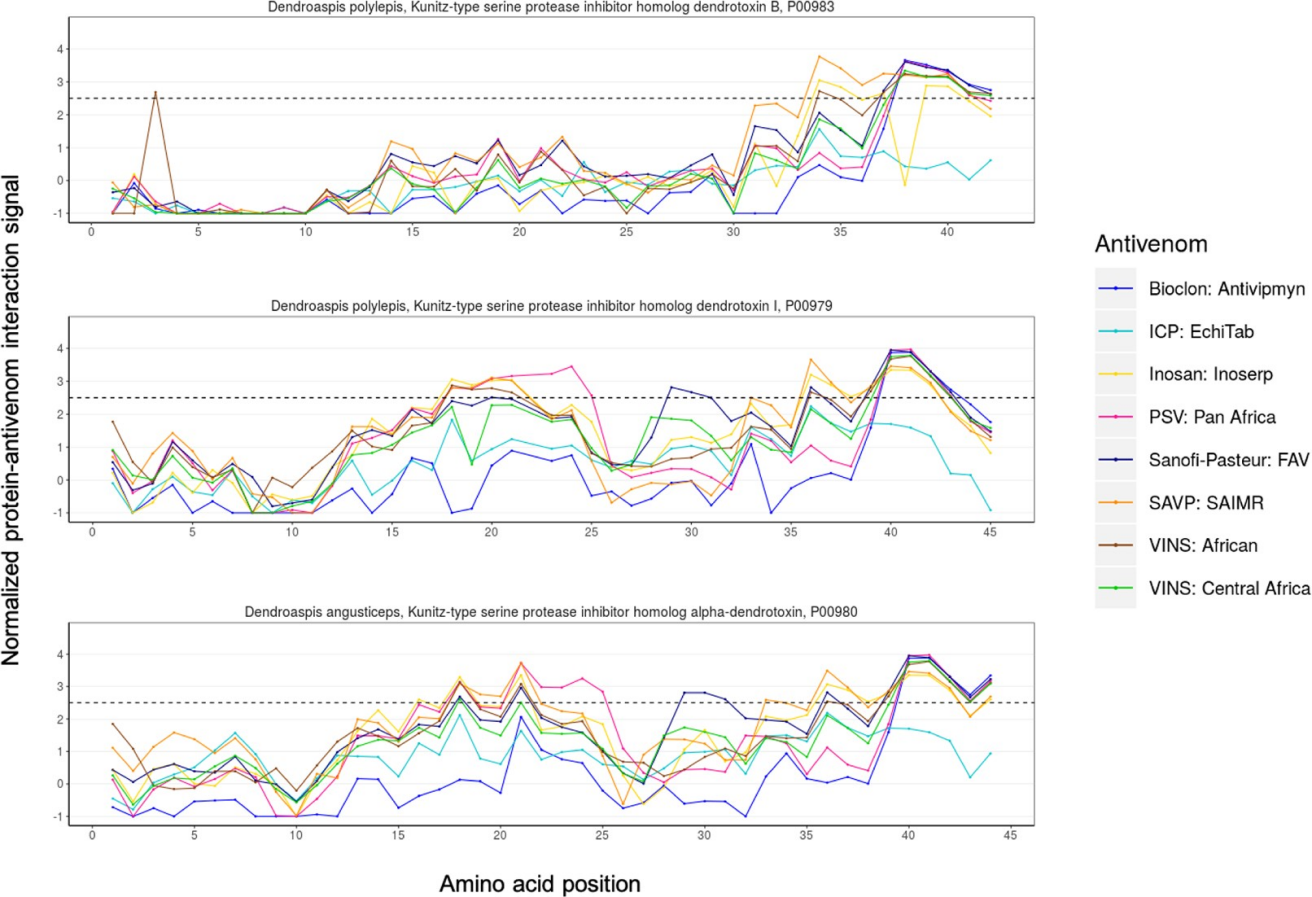
Dendrotoxin-antivenom interaction signals detected across all eight tested antivenoms.

We found that dendrotoxins from *D*. *polylepis* and *D*. *angusticeps* were poorly recognized by all antivenoms. PSV: Pan Africa presented the best, yet still poor, interaction signals against dendrotoxin I (P00979; *Dendroaspis polylepis*) and alpha-dendrotoxin (P00980; *D*. *angusticeps*; Fig 5). However, even though PSV: Pan Africa constitutes the only tested antivenom that included all four species of mambas in its immunizing mixture (*D*. *polylepis*, *D*. *angusticeps*, *D*. *jamesoni*, and *D*. *viridis*), the interaction signals against dendrotoxin I and alpha-dendrotoxin barely surpassed the significance threshold (Fig 5).

### 3FTxs: Cytotoxins

**Fig 6:**
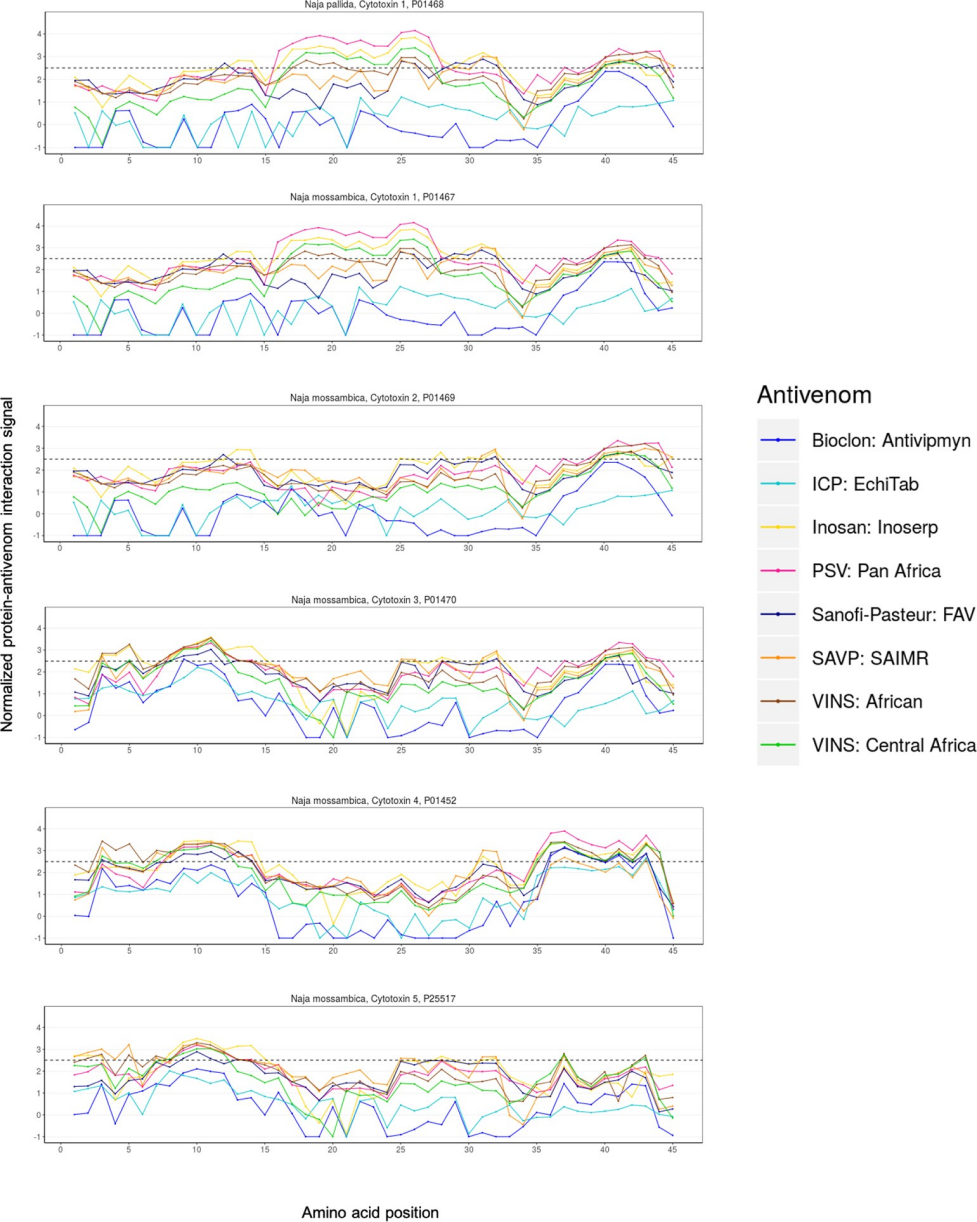
Cytotoxin-antivenom interaction signals detected across all eight tested antivenoms.

The interaction signals produced by antivenoms and six cytotoxins from two different spitting cobra species (*Naja pallida* and *N*. *mossambica*) were low, with cytotoxins 2, 3, and 5 (*N*. *mossambica*) indicating no signal at all (Fig 6). Cytotoxin 1 (P01468 and P01467) from both cobras and cytotoxin 4 (P01452) from *N*. *mossambica* presented signals above the significance threshold (Fig 6), despite neither of these venoms being used in the immunization process for the majority of the tested antivenoms. Notably, these data indicate that some snake venom cytotoxins are potentially conserved across cobras, whereas others are more species-specific.

**Fig 7:**
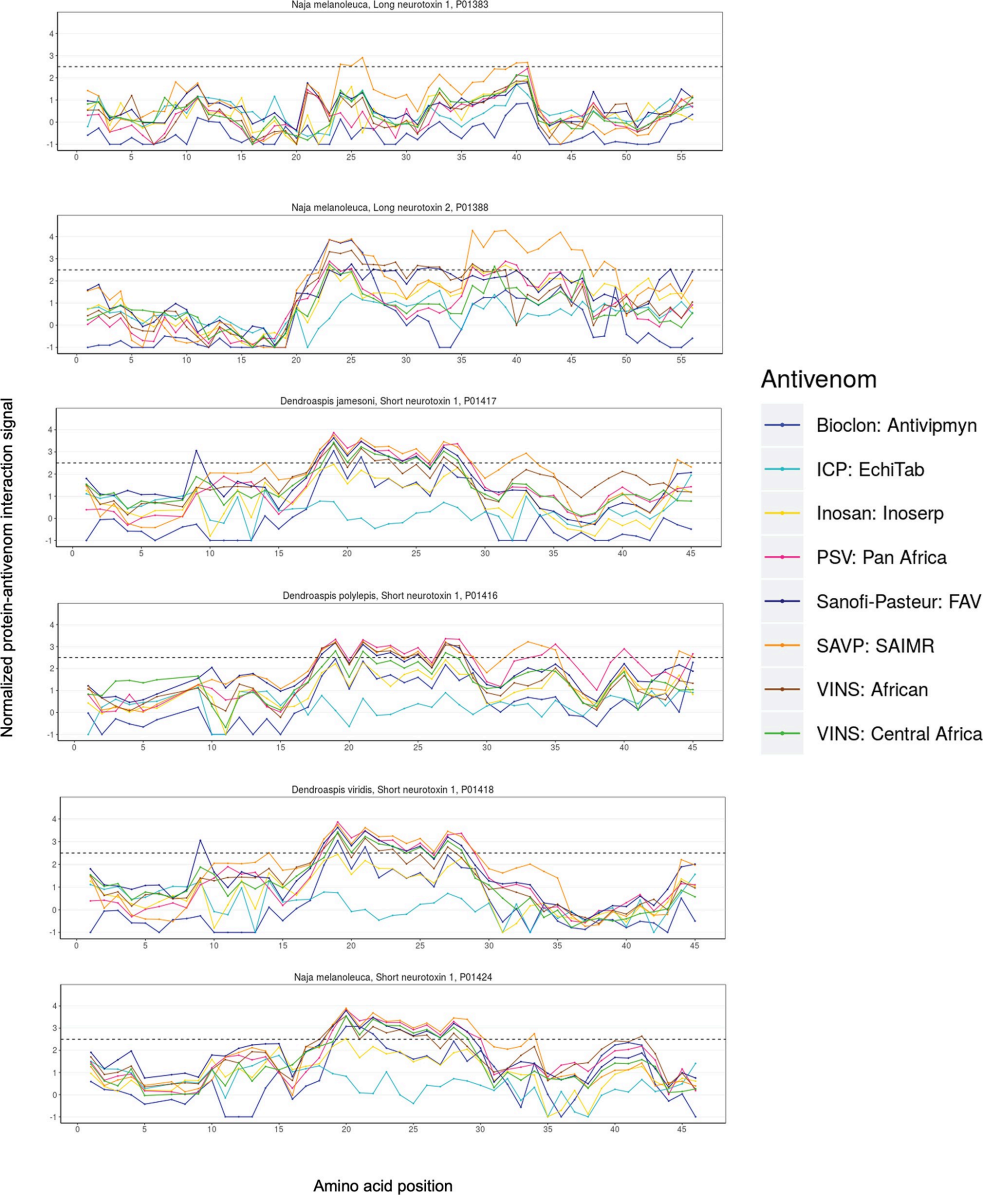
Neurotoxin-antivenom interaction signals detected across all eight tested antivenoms.

### 3FTxs: Long and short neurotoxins

Interaction signals between the eight antivenoms and both short and long neurotoxins were assessed in this study. The investigation of two long and four short neurotoxins from four different species of snakes (*Naja* and *Dendroaspis* spp.) against the various antivenoms indicated common epitopes across most of the short neurotoxins (15AA to 30AA; Fig 7); though, many of these sites barely surpassed the significance threshold. For the long neurotoxins, the only unique profile that we observed was between SAVP: SAIMR and long neurotoxin 2 (P01383) from *Naja melanoleuca* (Fig 7).

## Discussion

In this study we produced the most comprehensive venom-antivenom hdpm dataset to date [13–15], including eight polyspecific antivenoms and a peptide library derived from toxin protein sequences representing 40 snake species and 481 proteins from 23 protein families. Due to the expertise and time typically required to analyze data of comparable quantity [13–15], we have generated a free-to-use, user-friendly web application (STAB Profiles: http://tropicalpharmacology.com/tools/stab-profiles/) that enables easy access to all data generated in this study, in addition to all previously published venom-antivenom hdpm datasets. Through the use of the STAB Profiles web application, we were able to analyze the substantial amount of antibody-venom toxin binding profiles generated with relative ease, allowing rapid identification of specific venom-antivenom interactions simultaneously across different species of venomous snakes and diverse toxin families.

Our STAB Profiles web application allowed us to investigate to what extent different antivenoms interact with linear elements of epitopes from dominant and secondary toxin families [17]. We discovered that 3FTxs were the most bound toxin family out of all tested dominant toxins, with peptide interaction signals for phospholipase A_2_s (PLA_2_s) and metalloproteases (SVMPs) being markedly lower. The interaction with 3FTxs is of particular importance given their abundance, especially in elapids, and their high levels of toxicity [22]. In our study, we found that SAVP: SAIMR presented the highest peptide interaction signal coverage against this family of toxins. This is unsurprising, since it has been previously demonstrated that this antivenom displayed strong binding signals against 3FTxs when tested against whole venom from *Dendroaspis polylepis* [21], *Dendroaspis angusticeps* [19], and *Naja melanoleuca* [20]. Nevertheless, when tested *in vivo*, Lauridsen et al. found that lethality and overall toxicity were only neutralized at high anitivenom/venom ratios (e.g. 1.0 mg/mL in *D*. *angusticeps*); this indicated that even antivenoms with high peptide interaction signal coverage might not sufficiently neutralize 3FTxs [19–21]. These data present intriguing insights into the ability of antivenom antibodies to bind to specific toxin families.

We also aimed to identify linear toxin epitopic elements for antivenom antibodies across species and protein families. Whilst conformational epitopes could also be important to consider, linear epitopic elements are easier to reproduce and have been shown to often successfully approximate their true conformational epitopes [23–25]. The STAB Profiles web application can be used to rapidly identify potential linear epitopes and compare the epitope recognition ability of different antivenoms. One of the most notable findings we made is that antivenoms do not appear to recognize unique epitopes, and instead most seem to bind the same epitope, even when paraspecific. This immunodominance phenomenon is of particular interest when considering the development of targeted anti-toxin therapeutics that could be targeted towards the same epitopes.

Another finding with potential clinical implications, is the apparent lack or poor ability of some antivenoms to recognize peptides from key, medically relevant toxins (i.e. dendrotoxins (*Dendroaspis* spp.; [19, 21]), cytotoxins [26], short neurotoxins [19–21], and long neurotoxins [20]). We found that the majority of these toxins were very poorly recognized or not recognized at all, which is in line with reports from previous toxicovenomic studies that also suggest poor neutralization of these types of toxins [19–21, 27]. The reason for the poor recognition of these toxins by the antivenom antibodies on the hdpm could be caused by this group of toxins being neutralized via conformational epitopes. However, the lack of recognitions could also be linked to their low immunogenicity [28] and high diversity. Consequently, the high levels of toxicity of some of these toxins, together with the increasing evidence of poor recognizing and neutralizing capacity of antivenoms against these components, requires innovative approaches to improve the immunogenicity of low molecular weight toxins during manufacture. Fortunately, the use of neurotoxin-enriched venom mixtures for immunization [29] and recent advances towards next-generation therapeutics (e.g. recombinant oligoclonal antivenoms) might present some solutions for this problem, as well as help improve paraspecificity towards a wider range of venoms [18, 30, 31].

Whilst this study and the STAB Profiles web application have provided valuable new insights and will likely continue to do so in the future it is important to note that this approach has limitations. The conclusions that can be drawn from our web application are only as good as the data provided and while we aimed to ensure high standards throughout the data production and building of the web application, some venom toxins of certain species are better represented than others. Furthermore, batch-to-batch variations of the antivenoms could also distort some insights gained. Finally, the absence of a signal does not necessarily imply an inability to bind toxins, since binding might occur to conformational epitopes without strong linear epitopic elements. Indeed, we do not know the relative number of epitopes that encompass linear elements, large enough to be identified via hdpm. However, findings from previous studies indicate that the epitopes typically coincide with the functional sites of toxins [13]. Overall, we believe that as long as its limitations are considered, the STAB Profiles web application has the potential to play a significant role in understanding current antivenoms better, but also aid in the development of entirely new antivenom technologies.

## Conclusions

The considerable amount of venom toxin and antivenom antibody interaction data presented in this paper, in combination with the free and easy to use STAB Profiles web application, has the potential to be of substantial value to the venom-antivenom research community. The dataset and STAB Profiles web application have already helped gain insights into snake toxin epitopes and paraspecific binding capacities of African antivenoms. We encourage researches to use the data and STAB Profiles web application to help guide the improvement and optimization of current antivenoms for broader and increased therapeutic coverage. Furthermore, we believe that the data presented here will be crucial to the development and rapid assessment of next-generation broadly neutralizing antivenoms via the identification and characterization of relevant target epitopes for toxin neutralization.

## Methods

### Antivenoms and negative controls

Eight lyophilized polyspecific antivenoms were evaluated: (a) Antivipmyn Africa produced by Instituto Bioclon S.A. de C.V., Mexico City, Mexico (batch number DFB-150903, expiration date 09–2020) (**Bioclon: Antivipmyn**), (b) EchiTab-Plus-ICP produced by Instituto Clodomiro Picado (ICP), San José, Costa Rica (batch number 5370114PALQ, expiration date 01–2017) (**ICP: EchiTab**), (c) Inoserp Panafricain produced by Inosan Biopharma, S.A., Madrid, Spain (batch number 2VT08001, expiration date 08–2015) (**Inosan: Inoserp**), (d) Snake Venom Antiserum (Pan Africa) produced by Premium Serums and Vaccines Pvt. Ltd. (PSV), Maharashtra, India (batch number 062003, expiration date 01–2018) (**PSV: Pan Africa**), (e) FAV-Afrique produced by Sanofi-Pasteur SA, Lyon, France, (batch number K8453-1, expiration date 06–2016) (**Sanofi Pasteur: FAV**), (f) SAIMR (South African Institute for Medical Research) Polyvalent Snake Antivenom produced by South African Vaccine Producers (SAVP) (Pty) Ltd., Sandringham, South Africa (batch number BC02645, expiration date 07–2016) (**SAVP: SAIMR**), (g) Snake Venom Antiserum (African) produced by VINS Bioproducts Limited, Telangana, India (batch number 13022, expiration date 01–2018) (**VINS: African**), and (h) Snake Venom Antiserum (Central Africa) produced by VINS Bioproducts Limited, Telangana, India (batch number 12AS13002, expiration date 04–2017) (**VINS: Central Africa**). All antivenoms are of equine origin and a list of snake species they are meant to neutralize is provided in Table 1. A sample of naïve equine whole IgG purified from the plasma of horses that had not been immunized with venoms was obtained from Instituto Clodomiro Picado (ICP), San José, Costa Rica, and used as negative control. Prior to further use, all antivenom samples were diluted to the same protein concentration (22.17 mg/ml).

### *In silico* design of high-density peptide microarrays

A library of unique 16-mer peptides was designed *in silico* for microarray synthesis. The library comprised peptides derived from toxin protein sequences representing 40 snake species and 481 (447 Swiss-Prot reviewed) proteins representing 23 protein families. Protein fasta files were downloaded from the UniProt database, the sequences were trimmed for signal peptides and all unknown amino acid residues (X) were substituted by comparison with homologue protein sequences also included in the study. The 16-mer peptides were then constructed by splitting all toxins into *k-*mers of 16 amino acids by tiling at every amino acid position.

Furthermore, for all the peptides generated from secreted African snake venom toxin sequences, the library was extended to include peptides from an alanine scan substitution. The alanine scan was generated by substituting every second amino acid in each peptide with alanine as well as including a special alanine mutation peptide for 4 peptides to improve the dept of the data resulting in at least two substitutions for each residue, except the two terminal residues. The peptide library including the alanine scan finalized at a total of 164,255 peptides, which were then filtered to remove redundant peptides, resulting in a total of 163,254 unique peptides. A subset of 2,172 peptides were randomly selected from the library and replicated four times to check for correspondence in signal variances with the possibility of calculating the median of the standard deviation (SD) as a function of signal strength, which could in turn be used to calculate SD for peptide signals with only a single replicate. Lastly, an additional 1,001 completely random peptides were included in the library as negative controls for background peptide-antibody interaction noise, resulting in a total of 172,943 peptides included in each microarray. The final hdpm design was generated by assigning all peptides in the library random positions.

### Peptide microarray hybridization, sample binding, and processing

Three identical custom designed Roche NimbleGen 12-plex (12x135K) chamber microarray chips were synthesized by the Roche NimbleGen Peptide Lab in Madison, WI, USA. These three chips each included 12 identical microarray chambers, resulting in a total of 36 identical microarrays, which were synthesized with a Roche-NimbleGen Maskless Array Synthesiser (MAS). The MAS system uses light-directed solid-phase peptide synthesis, described as an amino-functionalized surface coupled with a 6-aminohexanoic acid linker and amino acid derivatives carrying a photosensitive 2-(2-nitrophenyl)propyloxycarbonyl (NPPOC) protection group. Amino acid coupling was performed for 3 minutes in dimethylformamide (DMF), using amino acids pre-activated with 2-(1H-benzotriazol-1-yl)-1,1,3,3,-tetramethyluronium hexafluorophosphate (HBTU) as an activator, hydroxybenzotriazole (HOBt) to suppress racemization, and *N*,*N*-diisopropylethylamine as base. The microarray was washed with *N*-methyl-2-pyrrolidone (NMP) following each coupling step. Site-specific cleavage of the NPPOC protection group was done by irradiation of an image created by a Digital Micro-Mirror Device (Texas Instruments, SXGA + graphics format), projecting light at a 365 nm wavelength. Coupling cycles were repeated to synthesize the full *in silico* generated peptide library. Treatment with 95% trifluoroacetic acid/4.5% water/0.5% triisopropylsilane for 30 min was used for final removal of side-chain protection groups.

All 12 microarray chambers on each of the three chips were incubated overnight at 4°C with chamber specific antivenom samples, one of the naïve equine serum negative control samples, or a pure binding buffer negative control sample. The first 12-plex chip had a microarray chamber with each of the 8 antivenom samples and the first naïve equine serum negative control sample mixed with binding buffer at a 1:250 dilution, two microarray chambers with the Sanofi Pasteur: FAV antivenom from at a 1:100 and 1:1000 dilution, respectively, and one microarray chamber with only the pure binding buffer negative control sample, resulting in a total fluid volume of 100 μL per microarray chamber. The second and third 12-plex chips had a microarray chamber with each of the 8 antivenom samples and the second naïve equine serum negative control sample at a 1:250 dilution, two additional microarray chambers with the second naïve equine serum negative control sample at a 1:2500 and 1:10000 dilution, respectively, and one microarray chamber with only the pure binding buffer negative control sample. This was followed by three washes, each of 10 minutes duration, with a Tris Buffered Saline and Tween 20 (TBST) buffer and incubation at room temperature for 3 hours with Alexa Flour 647-conjugated AffiniPure Goat Anti-Horse IgG (H+L) (Jackson ImmunoResearch Laboratories, Inc., PA, USA, code no. 108-605-003, lot no. 102264). After a final wash, the microarrays were dried and read using a MS200 microarray scanner, and signals were extracted using the NimbleGen DEVA signal extraction software, as also described by Engmark et al. [13].

### Signal normalization and classification of significant signals

Classification of peptides as binders or non-binders was based on the signal intensity. Before this classification, all signals were normalized with the purpose of determining a threshold for signal significance. First, signal values were cleaned for potential background noise. Evaluating one chip at a time, the position-specific signal observed in the sector evaluated with buffer as the primary antibody were subtracted from signals observed in sectors evaluating antivenom as primary antibody. This correction removed potential false-positive signals, as a result of non-specific secondary antibody binding. After cleaning of false positive signals, all peptide interaction signals were log transformed, resulting in a closer to normal distribution. For signal normalization the signals of the subset of 1,001 completely random peptides from all microarrays on every chip were used to calculate mean and standard deviation (SD) per chip. All non-random peptides per chip were then scaled by subtracting the calculated mean and dividing by the calculated SD. After normalization, peptide signals were represented by Z-score, and peptides with a normalized signal score higher than 2.5 (corresponding to a *p-*value of maximum 0.05) were considered significant. The 2,172 peptides present in replicates were represented by the median signal score, resulting in one signal score per peptide. All Perl scripts used in data treatment were made available through Mendeley data (http://dx.doi.org/10.17632/v88xfw5wyx.2), with the purpose of making our normalization strategy freely available to anyone wanting to work with the data in the future.

### Construction of Snake Toxin and Antivenom Binding Profiles (STAB Profiles)

Peptide to toxin affiliation was obtained by performing local alignment using BLASTp [32], for all peptides as well as a selection of toxin sequences representing the geographical region of Africa (305 proteins). The local alignment results were filtered and only alignments representing a 100% identity, no gaps, and an alignment length of 16 amino acids were considered in the further analysis (alanine scan results and sensitivity to mutations were not evaluated in this study, but have been included in the available dataset found at Mendeley data (http://dx.doi.org/10.17632/v88xfw5wyx.2; link also available in the STAB Profiles web application).

The binding profiles were constructed by assigning the interaction signal to the protein sequence position representing the beginning of the alignment. The position-specific score symbolized the interaction intensity for the following 16-amino acid peptide. As eight antivenoms were evaluated, all positions with a signal were represented by eight signal scores, each specific to the primary antivenom antibodies used in incubation.

Binding profiles were generated as diagrams of the 16-mer starting position in alignment of the total amino acid protein position on the x-axis and the normalized signal intensity on the y-axis. In this visualization, the full amino acid sequences are represented, and the observed interaction sites are illustrated as a peak in the graph. The entire data processing was performed using custom-built Perl scripts.

### Building the web application

The interactive web application was constructed using the R package “Shiny” [33] in RStudio v1.1.453 (RStudio Team, 2016) with R v3.5.0 (R Core Team, 2018). Shiny is used for turning analyses created with R into interactive web applications without the programmer requiring further knowledge of HTML, JavaScript, or CSS web programming. Shiny is intended to deliver interactive experiences that provide the end users with the option of changing input values on a webpage and having the results of an R program being written as output values back out to the web page in a reproducible way. It therefore allows the end user to have an interactive experience with the data available in the app in a simple and intuitive manner without the user being able to change the original data.

The STAB Profiles web application was built from three components; the user interface (UI) as a UI object controlling the layout and appearance of the application, a server function containing instructions needed to build the application, and a call to the “shinyApp” function, which combines the UI and server parts to create the Shiny web application object on the basis of the hdpm data described above. The UI object was designed to create the most intuitive and user-friendly interface for the application end user. The server function was built to subset the loaded snake toxin and antivenom binding data on the basis of the user’s selected input choices (antivenom(s), snake genus, snake species, protein family, protein sub-family, protein sub-sub-family) and visually display the subsetted data as one or more line-graphs in interactive plots using the R package “ggplot2” [34]. The interactive ggplots make it possible for the end user to choose specific points of interest on the graphs, which are then displayed in a HTML table widget as a downloadable csv-file using the “datatable” function from the “DT” [35] R package. The R package “dplyr” [36] was used for data wrangling and the subsetted data was also made available in a HTML table widget as a downloadable csv-file. Colors representing different antivenoms in the interactive plots were manually defined together with plot axes limits and a number of other visual specifications.
